# Therapeutic Efficacy of a Subunit Vaccine in Controlling Chronic *Trypanosoma cruzi* Infection and Chagas Disease Is Enhanced by Glutathione Peroxidase Over-Expression

**DOI:** 10.1371/journal.pone.0130562

**Published:** 2015-06-15

**Authors:** Shivali Gupta, Charity Smith, Sarah Auclair, Anahi De Jesus Delgadillo, Nisha Jain Garg

**Affiliations:** 1 Department of Microbiology and Immunology, School of Medicine, University of Texas Medical Branch, Galveston, Texas, United States of America; 2 Department of Pathology, University of Texas Medical Branch, Galveston, Texas, United States of America; 3 Institute for Human Infections and Immunity and the Sealy Center for Vaccine Development, University of Texas Medical Branch, Galveston, Galveston, Texas, United States of America; Albert Einstein College of Medicine, UNITED STATES

## Abstract

*Trypanosoma cruz*i-induced oxidative and inflammatory responses are implicated in chagasic cardiomyopathy. In this study, we examined the therapeutic utility of a subunit vaccine against *T*. *cruzi* and determined if glutathione peroxidase (GPx1, antioxidant) protects the heart from chagasic pathogenesis. C57BL/6 mice (wild-type (WT) and GPx1 transgenic (GPx^tg^) were infected with *T*. *cruzi* and at 45 days post-infection (dpi), immunized with TcG2/TcG4 vaccine delivered by a DNA-prime/Protein-boost (D/P) approach. The plasma and tissue-sections were analyzed on 150 dpi for parasite burden, inflammatory and oxidative stress markers, inflammatory infiltrate and fibrosis. WT mice infected with *T*. *cruzi* had significantly more blood and tissue parasite burden compared with infected/GPx^tg^ mice (n = 5-8, p<0.01). Therapeutic vaccination provided >15-fold reduction in blood and tissue parasites in both WT and GPx^tg^ mice. The increase in plasma levels of myeloperoxidase (MPO, 24.7%) and nitrite (iNOS activity, 45%) was associated with myocardial increase in oxidant levels (3-4-fold) and non-responsive antioxidant status in chagasic/WT mice; and these responses were not controlled after vaccination (n = 5-7). The GPx^tg^ mice were better equipped than the WT mice in controlling *T*. *cruzi*-induced inflammatory and oxidative stress markers. Extensive myocardial and skeletal tissue inflammation noted in chagasic/WT mice, was significantly more compared with chagasic/GPx^tg^ mice (n = 4-6, p<0.05). Vaccination was equally effective in reducing the chronic inflammatory infiltrate in the heart and skeletal tissue of infected WT and GPx^tg^ mice (n = 6, p<0.05). Hypertrophy (increased BNP and ANP mRNA) and fibrosis (increased collagen) of the heart were extensively present in chronically-infected WT and GPx^tg^ mice and notably decreased after therapeutic vaccination. We conclude the therapeutic delivery of D/P vaccine was effective in arresting the chronic parasite persistence and chagasic pathology; and GPx1 over-expression provided additive benefits in reducing the parasite burden, inflammatory/oxidative stress and cardiac remodeling in Chagas disease.

## Introduction

Chagas disease caused by *Trypanosoma cruzi* is endemic in Latin America and an emerging disease in the US and other developed countries. Overall prevalence of human *T*. *cruzi* infection is at ~16–18 million cases, and ~120 million people are at risk of infection [1]. Several years after infection, 30–40% of the infected individuals develop chronic cardiomyopathy with progressive irreversible tissue destruction, arrhythmia, thromboembolic events, and congestive heart failure [2], suggested to be associated with pathologic outcomes of persistence of parasite, inflammatory infiltrate, and oxidative stress in the heart [3,4].

The current knowledge on protective immunity and vaccine development efforts against *T*. *cruzi* [5] and the pathologic role of oxidative stress in Chagas disease [4,6] have recently been reviewed. Briefly, effective immune response for the control of *T*. *cruzi* infection requires activation of CD4^+^ and CD8^+^ T cells secreting Th1 cytokines, phagocytic activity of macrophages, lytic antibody response, and T lymphocytes cytotoxic activity [6]. Subsequently, several antigens, antigen-delivery vehicles, and adjuvants have been tested to elicit immune protection to *T*. *cruzi* in experimental animals (reviewed in [5,7–9]). We employed an unbiased computational approach for the identification of potential vaccine candidates [10] and through rigorous analysis over a period of several years, demonstrated that three candidate antigens (TcG1, TcG2, TcG4) were maximally relevant for vaccine development [10,11]. Co-delivery of these antigens as a prophylactic vaccine elicited greater immunity and protection from *T*. *cruzi* infection than was noted with individual candidate antigens [11–15]. Mice immunized with the DNA-prime/protein-boost vaccine constituted of TcG1, TcG2 and TcG4 were capable of controlling challenge infection as evidenced by a 90–97% decline in acute parasitemia and tissue parasite burden; and, subsequently, inflammatory infiltrate and tissue fibrosis were particularly absent in the heart and skeletal muscle of vaccinated mice [13].

We and others have reported that a pro-oxidant milieu is present in the myocardium in chronic Chagas disease [16,17]. Treatment with ROS scavengers or enhancement of the antioxidant capacity was beneficial in preventing myocardial oxidative adducts and hypertrophic responses and preserved the left ventricular function that otherwise was compromised in the chronic disease phase in experimental models [18,19]. A decline in oxidative stress in human Chagas disease patients given antioxidant supplement has also been shown [20,21]. These observations have supported the idea that the sustained oxidative stress contributes to pathologic outcome in Chagas disease.

In this study, we have sought to determine if therapeutic delivery of the vaccine along with enhancement of antioxidant status would be an effective treatment of chronic Chagas disease. C57BL/6 (wild-type (WT) and GPx1 transgenic (GPx^tg^) mice were infected with *T*. *cruzi*, and at the end of acute parasitemia, mice were given two-dose therapeutic vaccine, delivered by DNA-prime protein-boost (D/P) approach. We included in the therapeutic vaccine TcG2 and TcG4 antigens shown to elicit potent anti-parasite antibodies and CD8^+^T cell immunity [13,15]. GPx1 is a key enzyme for the cellular and mitochondrial defense against oxidative stress and it uses glutathione to reduce H_2_O_2_ and lipid peroxide [22]. Mice were harvested during chronic disease phase, and we examined whether the D/P therapeutic vaccine and/or enhanced antioxidant status were beneficial in controlling parasite persistence, chronic inflammation, and oxidative stress in chagasic disease.

## Materials and Methods

### Parasites and mice

*T*. *cruzi* trypomastigotes (SylvioX10/4 strain) were maintained and propagated by continuous *in vitro* passage in C2C12 cells. *T*. *cruzi* isolate and C2C12 cells were purchased from American Type Culture Collection (ATCC, Manassas VA). Mice over-expressing human glutathione peroxidase 1 (GPx-transgenic [GPx^tg^] have previously been described [23,24], and were kindly provided by Dr. R. Ann Sheldon, University of California San Francisco. The GPx^tg^ mice (CD1 background) were back-crossed with C57BL/6 WT mice for more than ten generations to generate GPx^tg^ mice on C57BL/6 genetic background. All animal experiments were performed according to the National Institutes of Health Guide for Care and Use of Experimental Animals. The protocol was approved by the Institutional Animal Care and Use Committee (IACUC) at the University of Texas Medical Branch, Galveston (Permit number: 805029).

### *T*. *cruzi* genes and generation of recombinant plasmids and proteins

Sequences for TcG2 and TcG4 (SylvioX10 isolate) have previously been submitted to Genbank (AY727915 and AY727917, respectively) [10,11]. *TcG2* and *TcG4* have been identified in CL Brenner sequence database, and exhibit 99–100% homology to XM_806323 and XM_816508, respectively. The cDNA for the two *T*. *cruzi* genes were cloned in eukaryotic expression plasmid pCDNA3.1 [10,11]. Plasmids encoding murine IL-12 (pcDNA3.msp35 and pcDNA3.msp40) and GM-CSF (pCMVI.GM-CSF) were previously described [11]. Recombinant plasmids were transformed into *E*. *coli* DH5-alpha competent cells, grown in L-broth containing 100-μg/ml ampicillin, and purified by anion exchange chromatography by using the Qiagen maxi prep kit (Qiagen, Chatsworth, CA).

The cDNAs for *TcG2* and *TcG4* were cloned in-frame with a C-terminal His-tag into a pET-22b plasmid (Novagen, Gibbstown, NJ). Plasmids were transformed in *BL21* (DE3) pLysS-competent cells, and recombinant proteins purified by using the poly-histidine fusion, peptide-metal chelation chromatography system [13]. After purification, proteins were exchanged out of elution buffer by dialysis, and we validated that LPS contamination in the proteins was <1.0 EU/ml determined by toxin sensor limulus amebocyte lysate (LAL) assay kit (GenScript Inc. Piscataway, NJ). All cloned sequences were confirmed by restriction digestion and sequencing at the Biomolecular Core Facility at UTMB.

### Infection and immunization

Mice (GPx^tg^ and WT littermates, 6-8-weeks old) were infected with *T*. *cruzi* (10,000 trypomastigotes per mouse, intraperitoneal). Forty-five days later, mice were immunized with the 1^st^ vaccine dose consisting of the TcG2- and TcG4-encoding plasmids with IL-12- and GM-CSF-expression plasmids (25-μg each plasmid DNA/mouse, intramuscularly). Twenty one days after the primary immunization, mice were given 2^nd^ vaccine dose constituted of recombinant proteins (TcG2 and TcG4, 25 μg of each protein emulsified in 5 μg saponin/100 μl PBS/mouse, intradermally). Mice were harvested at 150 dpi corresponding to chronic phase of disease development, and blood, sera/plasma, and tissue samples stored at 4°C and -20°C.

### Quantitative PCR and Real-time RT-PCR

For the measurement of parasite burden, blood DNA was isolated with a QiAamp Blood DNA mini kit (Qiagen, Chatsworth, CA). Skeletal muscle and heart tissue (50 mg) were subjected to proteinase K lysis, and total DNA was purified by phenol/chloroform extraction and ethanol precipitation. Total DNA (50 ng) was used as a template, and real-time PCR performed on an iCycler thermal cycler with SYBR Green Supermix (Bio-Rad, Hercules, CA) and *Tc18SrDNA*-specific oligonucleotides. Data were normalized to murine *GAPDH* and fold change in parasite burden (i.e. *Tc18SrDNA* level) calculated as 2^−ΔCt^, where ΔC_t_ represents the C_t_ (infected)—C_t_ (control) [19,25].

Total RNA from tissue samples (50 mg) was extracted using Trizol reagent (Invitrogen, Carlsbad, CA) and reverse transcribed using an iScript cDNA Synthesis Kit (Bio-Rad). First-strand cDNA was used as a template in a real-time PCR on an iCycler thermal cycler with SYBR Green Supermix (as above), and specific oligonucleotides were used for amplification of atrial natriuretic peptide (*ANP*) and brain natriuretic peptide (*BNP*) hypertrophy markers [26]. The threshold cycle (*Ct*) values for the target mRNAs were normalized to *GAPDH* mRNA, and the relative expression level of each target gene was calculated as above.

### Nitrate/nitrite content

The nitrite/nitrate content, indicative of NO production by iNOS, was monitored by the Griess reagent assay [27]. Briefly, plasma samples were diluted 1:5 with double distilled water. In 96-well plates, 50 μL plasma samples were mixed with 100 μl Griess reagent, consisting of 1% sulfanilamide in 5% phosphoric acid and 0.1% *N*-(1-napthyl) ethylenediamine dihydrochloride (1:1 ratio, vol/vol). After incubation for 10 min, formation of diazonium salt was monitored at 520 nm (standard curve: 2–100 μM sodium nitrite).

### Myeloperoxidase (MPO)

MPO activity was determined by a dianisidine-H_2_O_2_ method, modified for 96-well plates. Briefly, plasma samples (10-μg protein) were added in triplicate to 0.53 mM *o*-dianisidine dihydrochloride (Sigma, St. Louis, MO) and 0.15 mM H_2_O_2_ in 50 mM potassium phosphate buffer (pH 6.0). After incubation for 5 min at room temperature, the reaction was stopped with 30% sodium azide, and the change in absorbance was measured at 460 nm (ɛ = 11,300 M^−1^·cm^−1^) [28]. Results were expressed as units of MPO/mg protein, whereby 1 unit of MPO was defined as the amount of enzyme degrading 1 n mol H_2_O_2_ per min at 25°C.

### Antioxidant and oxidant levels

Heart and skeletal muscle tissue sections (50 mg) were washed with ice-cold Tris-buffered saline. Tissues were suspended in lysis buffer consisting of 50 mM Tris-HCl (pH, 7.5), 150 mM NaCl, 1 mM EDTA, 1 mM EGTA, 1% Nonidet P‐40, 2.5 mM KH_2_PO_4_, and protease inhibitor cocktail (tissue: buffer ratio, 1:10, w/v), and homogenized on ice using a Omni tissue homogenizer. Homogenates were centrifuged at 3000 *g* at 4°C for 10 min to remove cell debris and the homogenates were stored at −80°C [17]. Protein concentration was determined by Bio-Rad Protein Assay.

Total antioxidant capacity in tissue lysates was examined by using the Cayman Chemical Antioxidant Assay Kit (Ann Arbor, MI). Briefly, tissue homogenates (10 μg protein) were mixed in triplicate with 2,2'-azino-di-[3-ethylbenzthiazoline sulphonate] (ABTS, 150 μM) / metmyoglobin (2.5 μM) reagent, and the reaction was started with H_2_0_2_ (75 μM). The ability of antioxidants in the sample to inhibit the formation of ABTS•^+^ radical was monitored at 405 nm [29]. The capacity of the antioxidants in the sample to prevent ABTS oxidation was compared with that of Trolox, a water-soluble tocopherol analogue, and quantified as molar Trolox equivalents.

We measured advanced oxidation protein products (AOPPs) in tissue lysates by spectrophotometry [30]. Briefly, in 96-well plates, plasma samples (1:10 dilution in phosphate-buffered saline [PBS]; 200-μl/well) were mixed in triplicate with 10 μl of 1.16 M potassium iodide and 20 μl of 100% acetic acid. The formation of chloramine-T, which absorbs at 340 nm in the presence of potassium iodide, was immediately read at 340 nm using a M2 SpectraMax microplate reader (Molecular Devices, Sunnyvale, CA). A standard curve was prepared using chloramine-T (linear range, 1 to 100 μ mol, Sigma), and the AOPP concentration was expressed as μ mol chloramine-T equivalents.

### Histology

Tissue sections were fixed in 10% buffered formalin for 24 h, dehydrated in absolute ethanol, cleared in xylene, and embedded in paraffin. Five-micron sections of the heart and skeletal muscle tissue (≥ 3 tissue sections/organ/mouse, n = 4–6) were stained with hematoxylin/eosin (H&E) and Masson’s Trichrome [20] to examine inflammatory infiltrate/myocarditis and collagen deposition, respectively. Each tissue-section was analyzed for >10-microscopic fields (100 X magnifications) by two investigators who were blinded to identity of the experimental groups. Myocarditis (presence of inflammatory cells) from H&E stained sections was scored as 0 (absent), 1 (focal or mild, 0–1 foci), 2 (moderate, ≥2 foci), 3 (extensive inflammatory foci, minimal necrosis, and retention of tissue integrity), and 4 (diffused inflammation with severe tissue necrosis, interstitial edema, and loss of integrity) [26]. Inflammatory infiltrates were characterized as diffused or focal depending upon how closely the inflammatory cells were associated. The parasitic foci in skeletal muscle and heart tissue sections were scored as (0) absent, (1) 0–1 foci, (2) 1–5 foci, and (3)>5 foci. Cardiac fibrosis was assessed by measuring the Masson’s Trichrome-stained collagen area as a percentage of the total myocardial area using Simple PCI software (version 6.0; Compix, Sewickley, PA) connected to an Olympus polarizing microscope system (Center Valley, PA). All pixels with blue stain in Masson’s trichrome-stained sections were selected to build a binary image, subsequently calculating the total area occupied by connective tissue. Sections were categorized based on % fibrotic area: (0) <1%, (1) 1–5%, (2) 5–10%, (3) 10–15%, and (4) >15%.

### Statistical analysis

All experiments were conducted with triplicate observations per sample (n = 4–8 mice/group), and data are expressed as mean ± standard deviation (SD). All data were analyzed using InStat version 3 (GraphPad, La Jolla, CA), SPSS version14.0 (SPSS Inc., Chicago, IL), or SigmaPlot version 13.0 (Systat Software, San Jose, CA). Normally distributed data were analyzed by the Student’s *t* test (for comparison of 2 groups) and one-way analysis of variance (ANOVA) with Tukey's *post hoc* test (for comparison of multiple groups). Data sets that were found not to be normally distributed were analyzed with the Kruskal-Wallis test followed by the Mann-Whitney test to assess the differences between pair-wise comparisons. Significance is presented as ^#,*^ *p* <0.05 and ^##,**^ *p* <0.01 (*^,^**wild-type-versus-GPx^tg^; ^#,##^normal-versus-infected, or infected-versus-infected/vaccinated).

## Results

C57BL/6 wild‐type mice infected with 10,000 parasites exhibit peak parasitemia during 14–45 dpi, and develop chronic disease by ~120 dpi [15,25]. We employed this well‐established experimental model to examine the therapeutic efficacy of a 2-component D/P vaccine in controlling parasite persistence and chronic disease. Further, we included GPx1^tg^ mice in the study to determine if enhancing the cellular antioxidant status would alter the host’s resistance against chronic Chagas disease.

**Fig 1:**
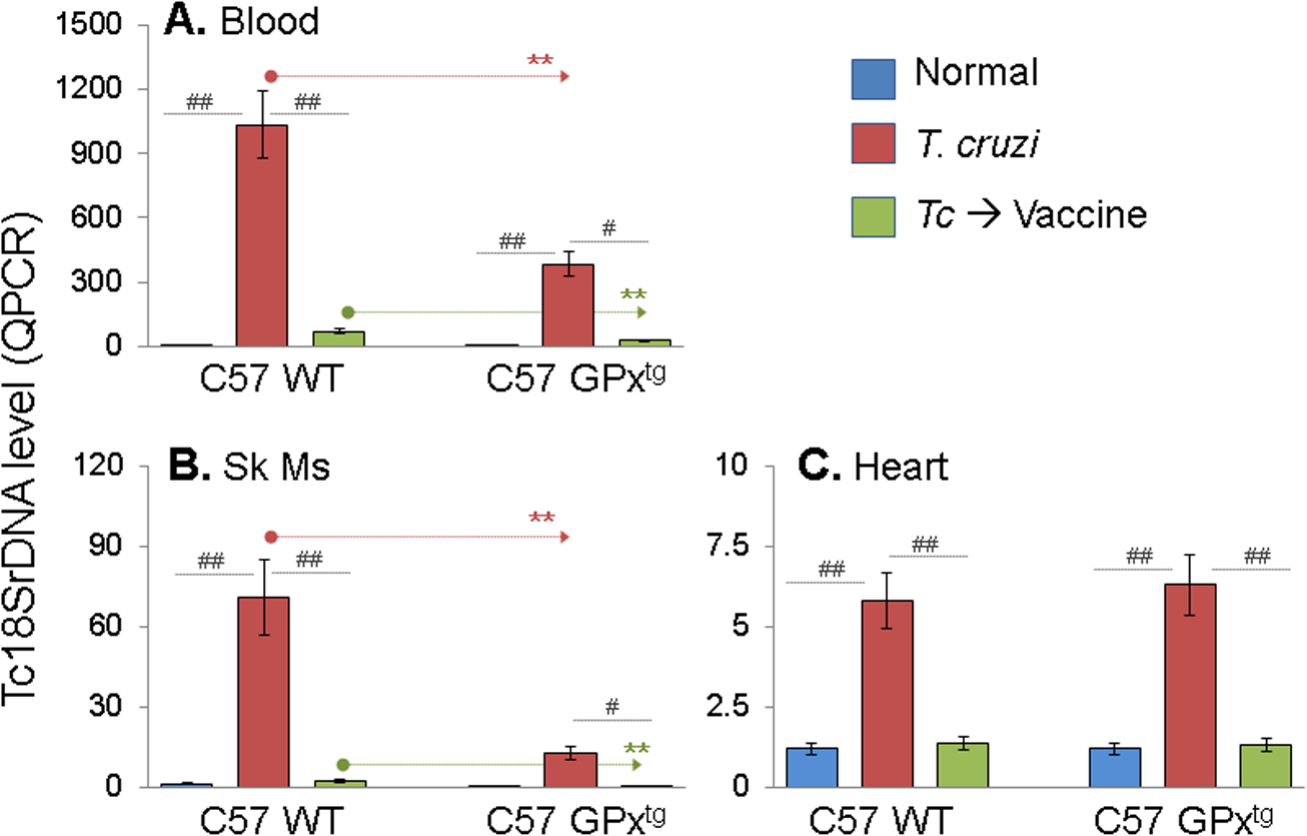
Control of *T. cruzi* persistence by a therapeutic vaccine in GPx-transgenic and wild-type mice. Glutathione peroxidase 1 over-expressing (GPx-transgenic, GPxt^g^) mice and wild-type (WT) littermates were infected with *T*. *cruzi* trypomastigotes (10,000 parasites/mouse), and immunized with a two-component DNA-prime/protein boost (D/P) vaccine (1^st^ dose: TcG2- and TcG4-encoding plasmids + IL-12- and GMCSF-expression plasmids at 45 days post-infection (pi); and 2^nd^ dose: recombinant TcG2 and TcG4 proteins at 66 days pi), as detailed in Materials and Methods. Mice were harvested at 150 days pi corresponding to the chronic disease phase. Total DNA isolated from blood **(A)**, skeletal muscle **(B)** and heart **(C)** of the infected/vaccinated and infected/non-vaccinated mice were subjected to quantitative real time PCR amplification for *Tc18SrDNA* sequence. Bar graphs show the *Tc18SrDNA* level normalized to murine *GAPDH* gene. In all figures, data are expressed as mean ± SD, and significance is presented as ^#,*^ *P* < 0.05 and ^##,**^ *P* < 0.01 (* wild-type versus GPx^tg^; ^#^ wild-type versus infected, or infected versus infected/vaccinated).

All mice, irrespective of the antioxidant status were infected by *T*. *cruzi*, evidenced by an increase in blood and tissue parasite burden (Fig 1). Therapeutic delivery of vaccine resulted in 14.7-fold and 29.5-fold decline in blood and skeletal muscle levels of *Tc*18SrDNA levels, respectively, in chronically-infected/vaccinated WT mice when compared to that noted in chagasic/non-vaccinated WT mice (Fig 1A & 1B, all ^#^p<0.05–0.01). Interestingly, chronically-infected GPx^tg^ mice exhibited 2.4–2.7-fold and 5.6-8-fold lower level of blood and skeletal tissue parasite burden, respectively, than was noted in infected/WT mice before as well as after therapeutic vaccine delivery (Fig 1A & 1B, **p<0.01). A similar decline in heart tissue parasite burden (4.2-fold) was observed in response to therapeutic vaccine in chronically-infected WT and GPx^tg^ mice (Fig 1C, ^##^p<0.01). Together, these data suggested that a) therapeutic D/P vaccine was efficacious in arresting parasite persistence in chagasic mice, and b) GPx^tg^ mice were better equipped than the WT litter-mates in controlling *T*. *cruzi* that resulted in enhanced efficacy of the therapeutic vaccine.

**Fig 2:**
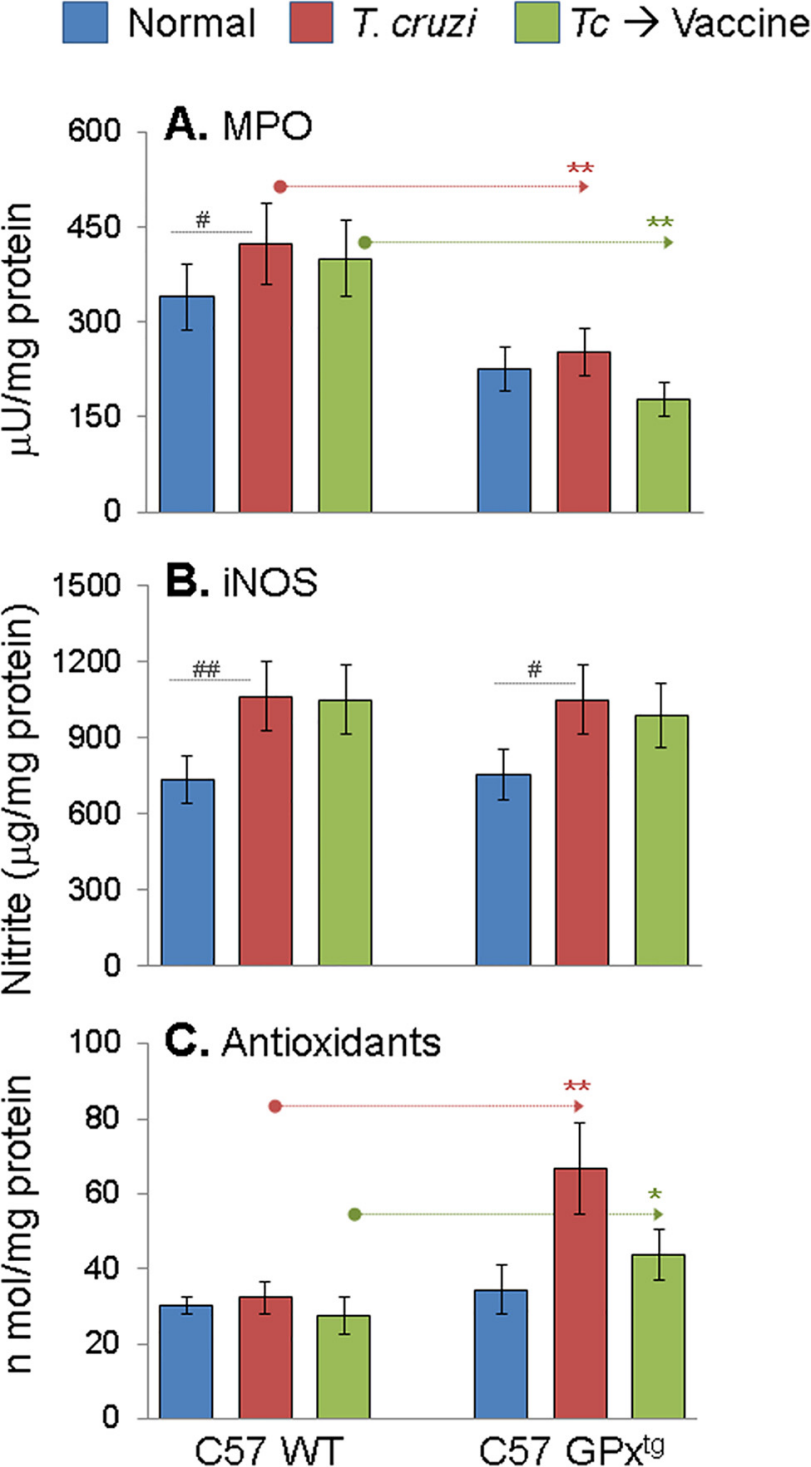
Inflammatory and antioxidant state in GPx^tg^ and WT mice during chronic Chagas disease (± therapeutic vaccine). Mice (GPx^tg^ and WT) were infected, vaccinated and harvested during chronic disease phase as in Fig 1. **(A)** Myeloperoxidase (MPO) activity in plasma by dianisidine-H_2_O_2_ method. **(B)** Nitrite content in reduced plasma samples, an indicator of inducible nitric oxide synthase (iNOS) activity, was determined by Griess reagent assay. **(C)** Total antioxidant capacity in heart homogenates of infected and infected/vaccinated mice was determined as detailed in Materials and Methods.

Oxidative and inflammatory stresses are known to be of pathologic significance in Chagas disease [6,31]. We evaluated plasma levels of MPO activity and nitrite content and myocardial antioxidant status to gain a quantitative measure of vaccine efficacy in controlling chronic stress in chagasic mice. The chronically-infected WT mice showed a 24.7% increase in plasma levels of MPO activity that was not significantly controlled after therapeutic vaccine delivery (Fig 2A, *p*<0.05–0.01). The GPx^tg^ mice exhibited a lower basal plasma level of MPO activity than was noted in WT mice. The MPO activity was only marginally increased post-infection (12% increase) and normalized to control level after vaccination in GPx^tg^ mice (Fig 2A). The plasma nitrite levels were increased by 45% and 39% in chronically-infected WT and GPx^tg^ mice, respectively, and remained high after therapeutic vaccination (Fig 2B). The basal level of oxidants (data not shown) and antioxidants (Fig 2C) were not different in WT and GPx^tg^ mice. In response to chronic infection, WT mice exhibited 3–4‐fold increase in myocardial oxidants, yet no increase in antioxidant levels was observed after infection or after vaccination-dependent control of chronic parasite persistence (Fig 2C). In comparison, GPx^tg^ mice exhibited significantly lower level of oxidative stress associated with >2-fold increase in antioxidant status during chronic infection when compared to that noted in chagasic WT mice (Fig 2C, **p<0.01). Therapeutic vaccination of chronically-infected GPx^tg^ mice was particularly effective and resulted in normal basal levels of myocardial oxidant (data not shown) and antioxidant status (Fig 2C). Together, the results presented in Fig 2 suggested that macrophage (NOS2/•NO) and neutrophil (MPO) activation contribute to chronic inflammatory state in chagasic mice and these responses were not subdued by therapeutic vaccine delivery. MPO, produced by activated neutrophils that use H_2_O_2_ and chloride to produce reactive hypochlorous acid, was not activated in chronically-infected GPx1^tg^ mice. Further, therapeutic vaccine mediated control of persisting *T*. *cruzi* did not prevent the antioxidant/oxidant imbalance in chagasic WT mice. However, the enhanced cellular antioxidant capacity in GPx^tg^ mice was beneficial in preventing myocardial oxidative stress caused by chronic *T*. *cruzi* infection.

**Fig 3:**
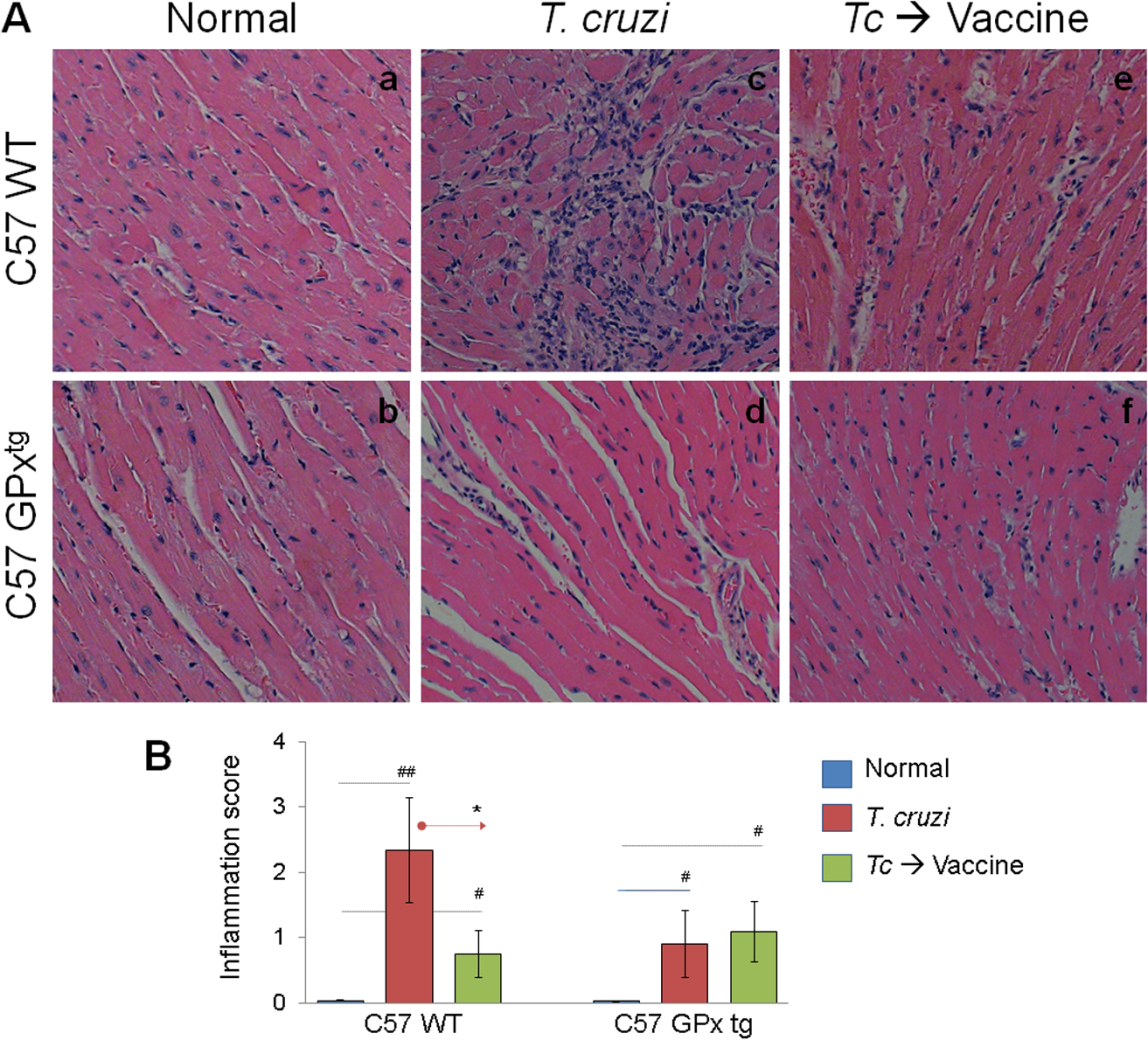
Myocardial inflammatory infiltrate in GPx^tg^ and WT mice during chronic Chagas disease (± therapeutic vaccine). Mice (GPx^tg^ and WT) were infected, vaccinated, and harvested during chronic disease phase as in Fig 1. **(A)** Paraffin-embedded left ventricular (LV) heart sections (5 μm) were examined by hematoxylin/eosin (H&E) staining (blue: nuclear; pink: muscle/cytoplasm). Shown are representative H&E images of tissue sections from normal (a&b), infected (c&d), and infected/vaccinated (e&f) mice harvested during chronic disease phase (>150 days post-infection, magnification: 20X). **(B)** The inflammatory score (myocarditis) were derived from two different experiments (n = 4 mice per group per experiment) as described in the Materials and Methods section.

**Fig 4:**
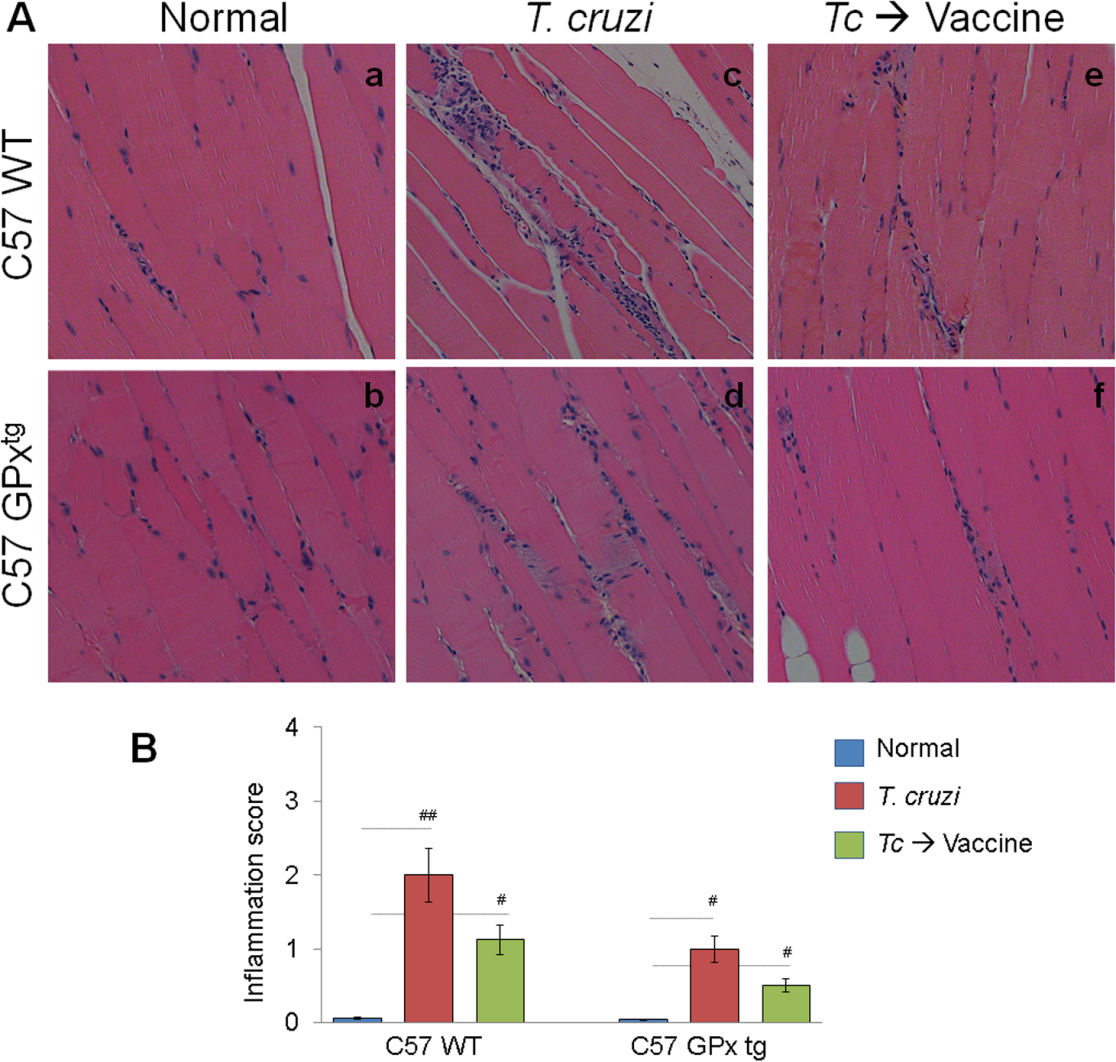
Chronic inflammation in skeletal muscle of GPx^tg^ and WT chagasic mice (± therapeutic vaccine). GPx^tg^ and WT mice were infected with *T*. *cruzi* and immunized with the two-component D/P vaccine as in Fig 1. Paraffin-embedded skeletal muscle sections (5 μm) were examined by hematoxylin/eosin (H&E) staining Shown are representative H&E images of tissue sections from normal (a&b), infected (c&d), and infected/vaccinated (e&f) mice harvested during chronic disease phase (>150 days post-infection, magnification: 20X). **(B)** The inflammatory score were derived from two different experiments (n = 4 mice per group per experiment).

Next, we determined the effects of therapeutic vaccine (± GPx over-expression) on *T cruzi–*induced myocarditis. Histological studies showed pronounced inflammatory infiltrate with diffused inflammatory foci in heart (score: 1–4, average: 2.3, Fig 3A.c) and skeletal muscle (score: 1–3, average: 2, Fig 4A.c) of chronically-infected WT mice. Myocardial degeneration with enlarged myocytes was particularly evident in chagasic WT mice. Therapeutic vaccination resulted in a substantial decline in myocardial (score: 0–2, average: 0.75, Fig 3A.e) and skeletal muscle (range: 0–3, average: 1.125, Fig 4A.e) levels of inflammatory infiltrate in chagasic WT mice. In comparison, chronically-infected GPx^tg^ mice presented a lower level of inflammatory infiltrate in heart (score: 0–3, average: 0.89, Fig 3A.d) and skeletal muscle (score: 0–2, average: 0.875, Fig 4A.d) that was further controlled after therapeutic vaccination (Fig 3A.f & Fig 4A.f). Together, the data presented in Figs 3 & 4 suggested that GPx^tg^ mice were better equipped than the WT mice in preventing the *T*. *cruzi*-induced inflammation of the heart, and therapeutic vaccination was effective in arresting the chronic infiltration of the inflammatory infiltrate in the heart in chagasic mice.

**Fig 5:**
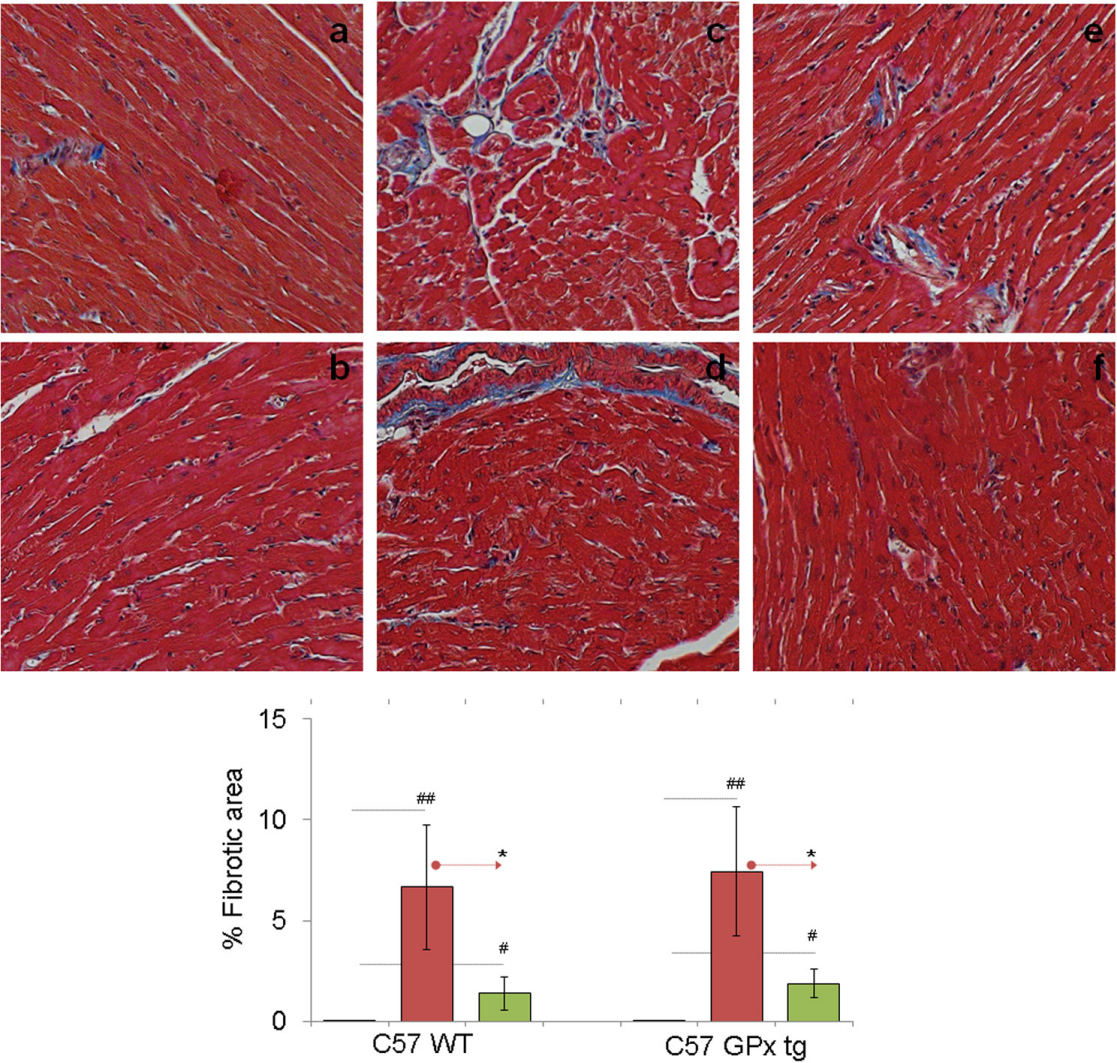
Cardiac fibrosis is controlled in GPx transgenic mice (± therapeutic vaccine). Mice (GPx^tg^ and WT) were infected and vaccinated as in Fig 1. Paraffin-embedded left ventricular (LV) heart sections (5 μm) were examined by Masson’s trichrome staining. Shown are representative images (collagen fibers: blue, nuclei: black, background: red) of tissue sections from normal (a&b), infected (c&d), and infected/vaccinated (e&f) mice harvested during chronic disease phase (>150 days post-infection, magnification: 20X). **(B)** The fibrosis score was derived from two different experiments (n = 4 mice per group per experiment), and was calculated as described in Materials and Methods section.

ROS and inflammatory mediators have been suggested to promote the development of interstitial and perivascular fibrosis, as well as myocardial hypertrophy. Histological staining with Masson's Trichrome of the tissue sections detected a high degree of myocardial remodeling associated with a significant disruption of cardiomyocytes in chronically-infected WT and GPx^tg^ mice (Fig 5). Semi-quantitative measurements of collagen area (blue, 3 regions per heart, n ≥ 4) suggested the fibrotic area, specifically around the vasculature, was significantly increased in chagasic WT (1.28–13.06%, average 6.65%) and GPx^tg^ (3.09–16.52%, average 7.42%) mice when compared to that noted in normal mice (compare Fig 5A.a&b with Fig 5A.c&d, p<0.01). The increase in collagen deposition was associated with up to 50% increase in mRNA levels for ANP and/or BNP hypertrophy markers in the myocardium of chronically infected WT and GPx^tg^ mice (*p < 0.01). Therapeutic vaccination resulted in a notable decline in myocardial collagen area in WT (0.29–4.79%, average 1.39%) and GPx^tg^ (0.486–3.658%, average 1.87%) chagasic mice (compare Fig 5A.c&d with Fig 5A.e&f). These data suggested that therapeutic vaccination was beneficial in controlling the chronic evolution of hypertrophic and fibrotic responses in chagasic mice, and GPx1^tg^ mice may be marginally better in preventing the chagasic cardiac remodeling.

## Discussion

Before setting the goal of new therapy development, an important question is whether it will fill gaps in achieving health benefits and if it will be an economically viable approach. Several studies, including our published reports (reviewed in [5]), have shown the prophylactic subunit vaccine mediated control of infection and disease in experimental models generally resembles that noted in the 60–70% of the chagasic patients that remain seropositive and maintain residual parasites for their entire lives, but do not develop a clinically symptomatic form of the disease [3]. In terms of treatment, acutely-infected patients, irrespective of their ages, are shown to respond to treatment with the anti-parasite drug benznidazole and be cured, defined by the control of acute parasitemia and myocarditis [32]. However, benznidazole and nifurtimox (anti-trypanosomal drugs) have not shown efficacy in the treatment of indeterminate status or chagasic cardiomyopathy [33]. The intolerance and unacceptable side effects in 30–50% of the treated individuals [34], mutagenic properties, and contra-indication in pregnancy have restricted the use of these drugs as a standard therapy in chagasic patients [35]. An effective therapeutic vaccine for human Chagas disease could prevent cardiac complications among the estimated 40,000 new cases of Chagas disease that occur in Latin America annually, avert over 600,000 DALYs annually that result from cardiomyopathy and gastrointestinal disease, and prevent 10,000 deaths or more annually [36]. Computer simulation modeling studies suggest that even when the risk of infection is only 1% and protective efficacy is only 25%, a vaccine would be economically viable and beneficial as long as the cost is US$20 or lower per vaccine dose [37]. The potential savings in term of cost of treatment per patient-year are estimated to be US$1028, with lifetime costs averaging US$11,619 per patient [38]. Thus, we believe that vaccination to reduce the frequency and severity of clinical disease by decreasing the extent of persistent parasite burden is urgently needed to improve the health outcomes in chagasic patients; and continuing efforts towards developing a prophylactic and therapeutic vaccine against *T*. *cruzi* infection and Chagas disease are economically justifiable.

Based upon several studies that we have conducted, we believe TcG2 and TcG4 candidate antigens are an excellent choice for therapeutic vaccine development, and a heterologous prime/boost approach for vaccine delivery is highly efficacious against *T*. *cruzi* and Chagas disease. The selected candidates TcG2 and TcG4 tested in this study are highly conserved in clinically relevant *T*. *cruzi* strains, and expressed (mRNA/protein) in infective trypomastigote and intracellular amastigote stages of *T*. *cruzi* [10]. We have shown the delivery of candidate antigens as a DNA-prime/protein-boost or DNA-prime/MVA-boost preventative vaccine was highly effective in generating protective immunity consisting of parasite- and antigen-specific lytic antibodies and type 1 CD8^+^ cytotoxic T lymphocytes against challenge infection and chronic disease in mice [14,15]. The enhanced efficacy of a heterologous prime/boost approach for vaccine delivery could be because delivery of antigens as DNA vaccine elicits robust T-cell responses, which are critical for the development of T-cell-dependent antibody responses [39,40]. Delivery of vaccine candidates as recombinant proteins is generally more effective at eliciting antibody responses and may directly stimulate antigen-specific memory B cells to differentiate into antibody-secreting cells, resulting in production of high-titer, antigen-specific antibodies [41,42]. Therefore, DNA-prime plus protein-boost is a complementary approach that overcomes each of their respective shortcomings. This, to the best of our knowledge, is the first study demonstrating the therapeutic efficacy of a subunit vaccine delivered by a DNA-prime/protein-boost approach in arresting *T*. *cruzi* persistence and chagasic disease in a murine model. We vaccinated mice during the indeterminate state when, similar to humans, the acute parasitemia was controlled and clinical disease symptoms have not yet developed. A majority of human patients are generally identified during this phase to be seropositive and carrying *T*. *cruzi* infection. Importantly, therapeutic delivery of TcG2/TcG4-encoding DNA-prime/protein-boost vaccine arrested the progression to chronic disease phase, evidenced by up to 10-fold control of peripheral and tissue levels of parasite burden as determined by a highly sensitive qPCR approach (Fig 1), and a significant decline in tissue infiltration of inflammatory infiltrate (Figs 3 & 4), and cardiac remodeling (Fig 5) that were otherwise pronounced in WT infected/non-vaccinated chagasic mice. Others have shown that immunotherapy with a Tc24- or TSA1-encoding DNA vaccine immediately after lethal infection led to survival of >70% of mice [43] and when given after acute parasitemia led to reduced cardiac inflammation [43,44] in infected mice. We surmise that therapeutic vaccines targeting *T*. *cruzi* parasites provide a relevant approach for reducing the cardiac tissue damage in Chagas disease.

We included mice overexpressing GPx in this study to test the idea that these mice will be better equipped in controlling chagasic pathology. Our observation of a low grade inflammation and a decline in the expression of hypertrophic markers and collagen deposition in chronically-infected/GPx^tg^ mice suggests that ROS signals inflammatory responses and hypertrophic remodeling in chagasic myocardium. Indeed, ROS is known to signal inflammatory responses in diverse disease models and both ROS and inflammatory cytokines, e.g., TNF-α, IL-1β, and MCP-1, can promote fibrosis and tissue remodeling. The treatment with an antioxidant or enhanced mitochondrial antioxidant capacity is shown to result in a significant decline in myocardial oxidative adducts concurrent with preservation of a cardiac hemodynamic state in chagasic rodents [19,45]. A decline in oxidative stress in human Chagas disease patients given Vitamin A is also shown [20]. These observations, thus, support the idea that sustained oxidative stress is of pathological importance in chagasic cardiomyopathy, and antioxidants should be considered as adjunct therapies along with anti-parasite treatments in arresting chronic chagasic pathology.

Surprisingly, we observed a decline in peripheral and tissue parasite burden in GPx^tg^ mice with or without therapeutic vaccine delivery, suggesting that oxidative state of the host determines the quality and efficacy of immune responses in arresting parasite survival and persistence. A recent study has shown a slower increase in blood parasitemia in mice given antioxidant (vitamin C) therapy, though authors noted no significant differences in tissue levels of *T*. *cruzi* and inflammatory infiltrate in chronic disease phase [46]. GPx deficiency has been linked to mutation of coxsackie virus B3 viral genome, with replacement of G base (most vulnerable to oxidation) at three of the seven sites [47], and enhanced myocarditis in Gpx1^-/-^ mice, though authors did not conclude enhanced viral burden. Likewise, GPX1^-/-^ mice developed higher levels of influenza A virus induced lung inflammation but it was not associated with increased viral load [48]. Thus, our observation of a better control of parasite burden in GPx^tg^ chagasic mice provide the first indication of a role of oxidative state of the host in eliciting appropriate immune responses. How ROS may alter host immunity in the context of *T*. *cruzi* infection and Chagas disease is not known. Besides signaling of NF-κB pathway of cytokine gene expression, ROS is suggested to enhance proliferation and activation of proinflammatory immune cells via activation of glycolytic metabolic pathways. However, over-production of ROS can alter the function of immune cells by direct oxidative damage or oxidative inhibition of transcription factors and cell-surface receptors involved in activation of adaptive immunity. Future studies delineating the kinetics of ROS signaling of metabolic pathways for altering the pro-inflammatory function of immune cells will help design novel strategies for achieving pathogen control [49,50] versus controlling chronic inflammatory state as is suitable for diverse heart diseases.

In summary, we have shown the therapeutic utility of a subunit vaccine against *T*. *cruzi* demonstrated in WT and GPx^tg^ mice. Our data showed that therapeutic vaccination provided >15-fold reduction in blood and tissue parasites, and subsequently, chronic myocarditis was controlled in mice receiving therapeutic vaccine. The Gpx^tg^ mice were better equipped than the WT mice in arresting the chronic parasite persistence, inflammatory/oxidative stress and cardiac remodeling in Chagas disease.
